# Supplementary figures and images for: Host Carbon Dioxide Concentration Is an Independent Stress for Cryptococcus neoformans That Affects Virulence and Antifungal Susceptibility
Source: mBio. 2019 Jul 2;10(4):e01410-19. doi: 10.1128/mBio.01410-19 (PMC6606813; doi:10.1128/mBio.01410-19)

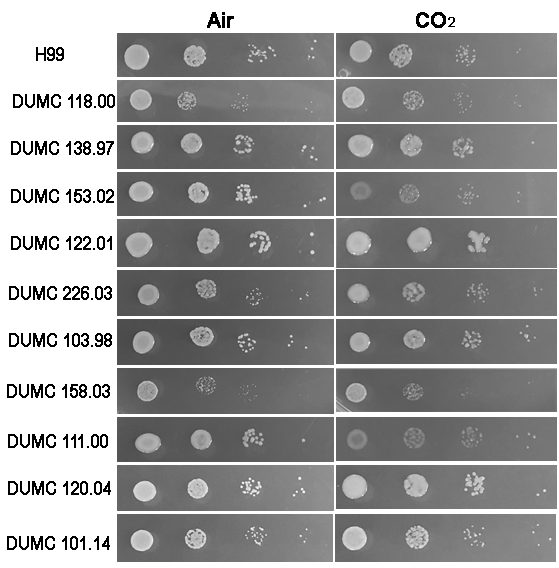

Supplement: FIG S1 [file mBio.01410-19-sf001.tif]

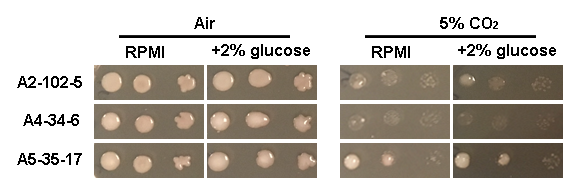

Supplement: FIG S2 [file mBio.01410-19-sf002.tif]

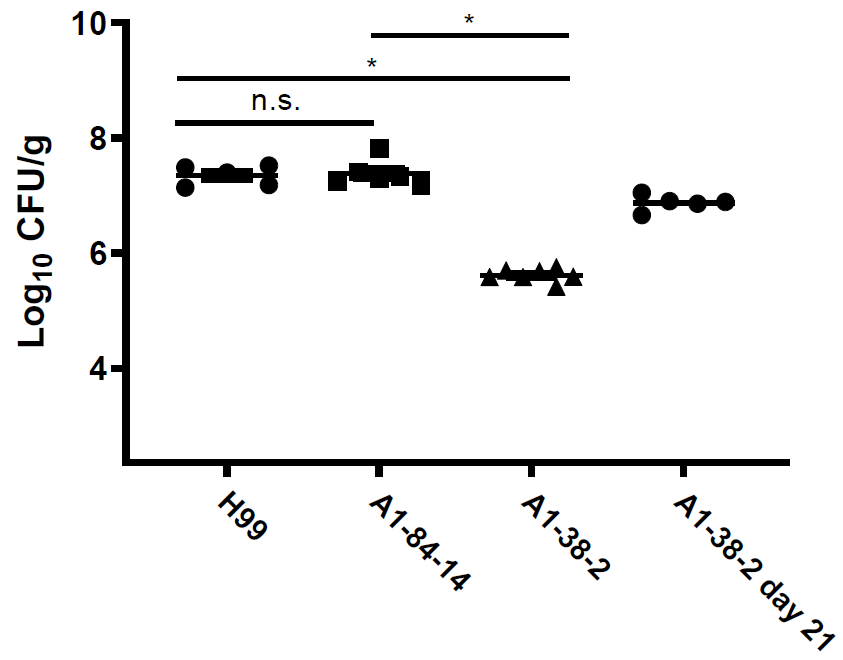

Supplement: FIG S3 [file mBio.01410-19-sf003.tif]

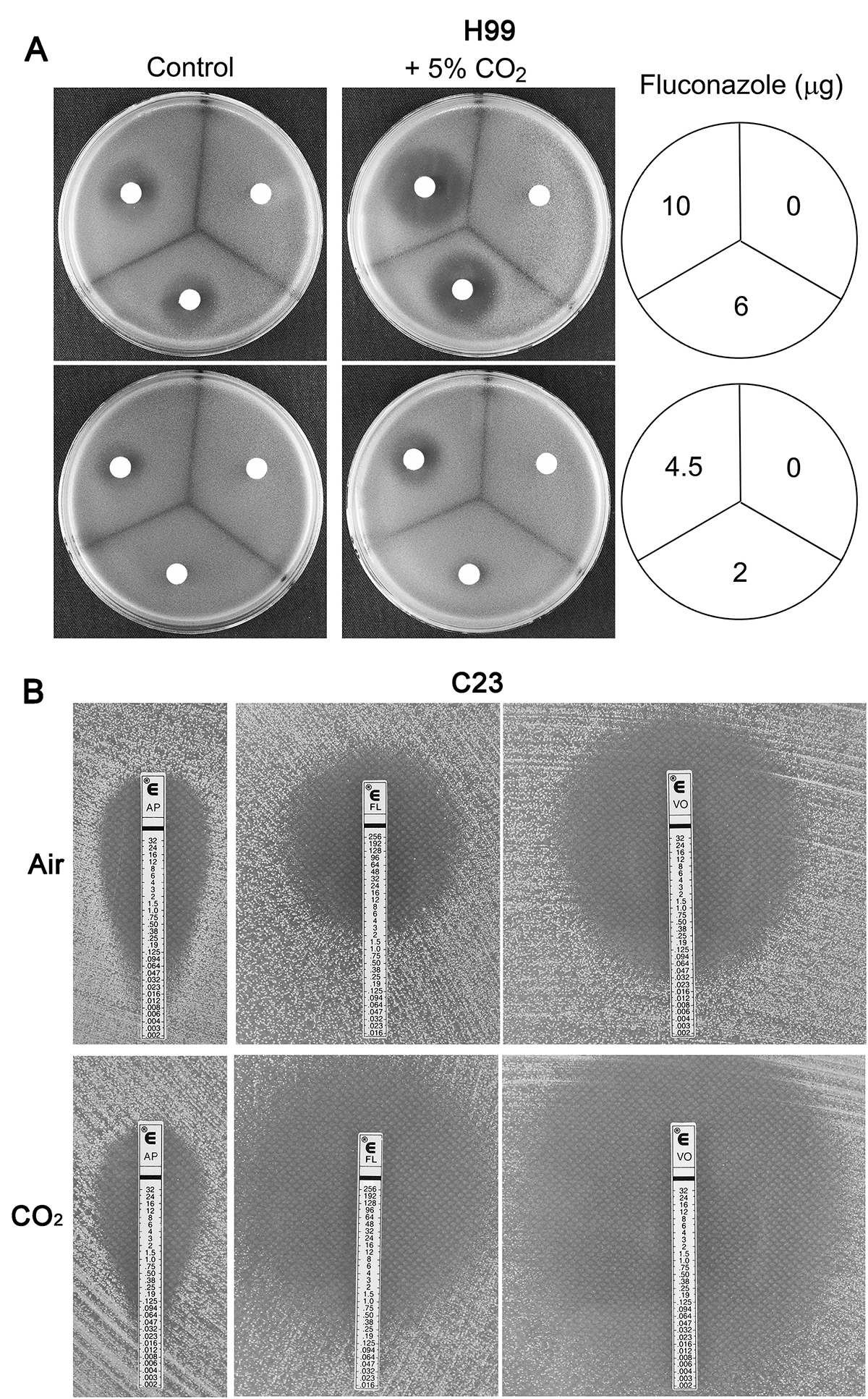

Supplement: FIG S4 [file mBio.01410-19-sf004.tif]
